# LC-ToF-ESI-MS Patterns of Hirsutinolide-like Sesquiterpenoids Present in the *Elephantopus mollis* Kunth Extract and Chemophenetic Significance of Its Chemical Constituents

**DOI:** 10.3390/molecules26164810

**Published:** 2021-08-09

**Authors:** Gabin Thierry M. Bitchagno, Jean Garba Koffi, Ingrid Konga Simo, Donald Ulrich K. Kagho, Augustin Silvere Ngouela, Bruno Ndjakou Lenta, Norbert Sewald

**Affiliations:** 1 Department of Chemistry, Faculty of Sciences, University of Dschang, Dschang, Cameroon; simoingrid@yahoo.fr (I.K.S.); sngouela@yahoo.fr (A.S.N.); 2 Organic and Bioorganic Chemistry, Faculty of Chemistry, Bielefeld University, 33501 Bielefeld, Germany; norbert.sewald@uni-bielefeld.de; 3 Department of Chemistry, Higher Teacher Training College, University of Yaoundé I, P.O. Box 47 Yaoundé, Cameroon; garbakoffijean@yahoo.fr (J.G.K.); lentabruno@yahoo.fr (B.N.L.); 4 Department of Organic Chemistry, Faculty of Science, University of Yaoundé 1, P.O. Box 812 Yaoundé, Cameroon; dkaghokenou@yahoo.fr

**Keywords:** LC–MS, Asteraceae, sesquiterpenoids, lactones, cytotoxicity, antimicrobial activity

## Abstract

A total of nine sesquiterpenoid lactones together with phenolic compounds and other terpenes were identified from the crude methanol extract of *Elephantopus mollis* Kunth. Compounds were isolated using different chromatographic techniques and their structures were determined by NMR and IR spectroscopy as well as mass spectrometry. The structures of some detected compounds were assigned based on LC-ToF-ESI-MS screening of main fractions/subfractions from flash chromatography and comparison with isolated analogues as standards. The findings revealed not only the in-source loss of water as the base peak in hirsutinolides but also the in-source loss of corresponding alcohol when the oxygen at position 1 is alkylated. The present study also draws up a complement of data with respect to hirsutinolide-like sesquiterpene lactones whose LC-MS characteristics are not available in the literature. The chemophenetic significance is also discussed. Some of the isolated compounds were reported for the first time to be found in the species, the genus as well as the plant family. The medium-polar fractions of the crude extract, also containing the larger amount of sesquiterpenoid lactones, exhibited activity both against a cancer cell line and bacterial strains. Isolated lactones were also active against the cancer cell line, while the chlorogenic derivatives also valuable in *Elephantopus* genus showed potent radical scavenging activity. This is the first report of cytotoxic and antibacterial activities of our samples against the tested strains and cell line. The present study follows the ongoing research project dealing with the characterization of taxa with antibacterial and antiparasitic activities from Cameroonian pharmacopeia.

## 1. Introduction

*Elephantopus mollis* Kunth is a plant used for primary healthcare since ancient times. It belongs to Asteraceae and constitutes together with roughly 31 other species the group *Elephantopus* [1]. *E. mollis* and *E. scaber* are the most popular species of the genus [2,3]. They have been screened for their pharmacological and phytochemical profiles [1,4,5]. *Elephantopus* species are sources of a class of sesquiterpenoid lactones named elephantopus-type lactones with a furane ring (C-2/C-5) in germacranolides [1,2,3,4,5,6]. They also produce glaucoside- and guaianolide-type lactones even in less amount alongside phenolic compounds and other terpenes [1,4,5,7]. Sesquiterpene lactones have been proven to be the active principle of the plant against various diseases [1,4,5,7].

However, the efficacy of a plant in ethnopharmacology might be highly depending on the area of collection [8]. In addition, during the chemical profiling by chromatography, low abundant compounds in the extract are most of the time lost, limiting the standardization of the plant as phytodrug. To solve such problems, efforts have been focused on highly sensitive methods such as mass spectrometry (MS), which can help to identify most of the constituents of an extract. Pure compounds isolated from the studied plant could alternatively help to establish the MS fingerprint of this group of chemicals [9]. These standards could then provide an almost exhaustive chemical profile of a taxa [9]. Accordingly, few reports are available in the literature on LC-MS characteristics of different sesquiterpenoid groups found in *Elephantopus* genus [9]. In our attempt to elucidate the cytotoxicity and antibacterial properties of *E. mollis*, we isolated and identified/detected reported sesquiterpenoid lactones by LC-MS. The results could be relevant in view of formulating the plant extract to relieve pains and diseases in less developing countries where the use of traditional medicine still prevails. The present study follows the ongoing research project dealing with the characterization of taxa with antibacterial and antiparasitic activities from Cameroonian rain forests and pharmacopeia [8,10,11,12].

## 2. Results

**Figure 1:**
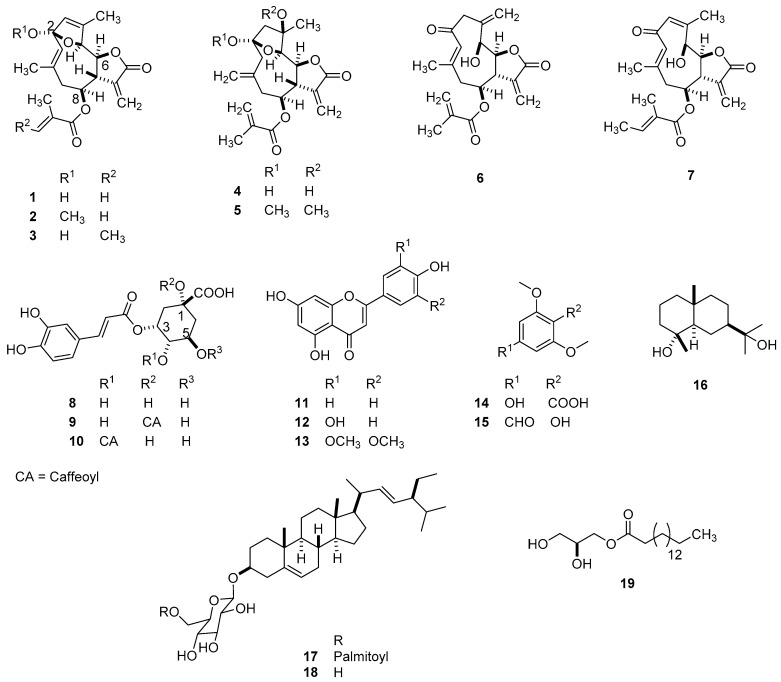
Structures of isolated compounds **1**–**19.**

Compounds (**1**–**19**) were isolated chromatographically, their structures were established using NMR and MS techniques and compared to related compounds in the literature (Figure 1). 

This includes 2-deethoxy-2*β*-hydroxyphantomolin (**1**) [5], 2*β*-methoxy-2-deethoxyphantomolin (**2**) [13], 2-deethoxy-8-*O*-deacylphantomolin-8-*O*-tiglinate (**3**) [14], 8-*O*-methacryloylelephantane (**4**) [5], 2,4-bis-*O*-methyl-8-*O*-methacryloylelephantane (**5**), molephantin (**6**), molephantinin (**7**) [15], isochlorogenic acid (**8**) [16], 1,5-dicaffeoylquinic acid (**9**) [17], 4,5-dicaffeoylquinic acid (**10**) [17], apigenin (**11**) [18], luteolin (**12**) [19], tricin (**13**) [20], 2,6-dimethoxy-4-hydroxybenzoic acid (**14**) [21], 4-formylsyringol (**15**) [22], cryptomeridiol (**16**) [23], *β*-sitosterol-3-*O*-*β*-D-glucopyranoside-6′-*O*-palmitate (**17**) [24], *β*-sitosterol-3-*O*-*β*-D-glucopyranoside (**18**) [24] and glycerine monopalmitate (**19**) [24].

### 2.1. LC–MS Analysis of Fractions for Structure Identification

**Figure 2:**
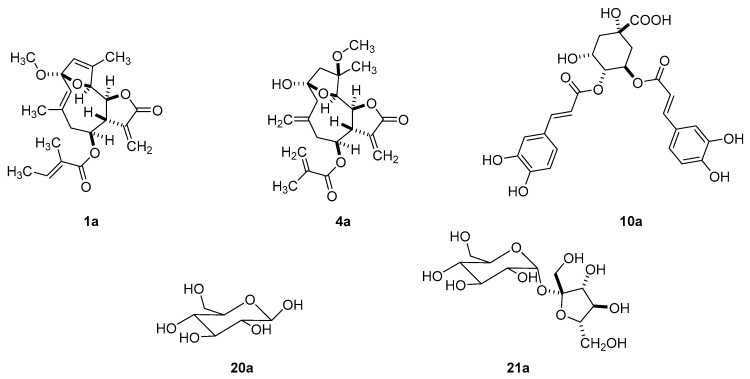
Dereplicated compounds in *E. mollis* whole crude methanol extract.

A non-exhaustive analysis of the LC-ToF-ESI-MS profiles of main middle-polar fractions from flash chromatography of the methanol extract led to the detection of further sesquiterpene lactones (**1a** and 4**a**), chlorogenic acid derivatives (**10a**) and carbohydrates (**20a**–**21a**) (Figure 2). 

Da Silva et al. (2020) reported the ESI-QToF-MS characterization of hirsutinolide- and glaucolide-type sesquiterpenoid lactones, the two sub-classes isolated in our study [9]. As a result, hirsutinolide skeletons give rise to an extremely low abundance of the quasi-molecular ion [M + H]^+^ (2–10% RA) and sodium adduct [M + Na]^+^ (10–20% RA) while the base peak is the in-source water loss ion [M + H − H_2_O]^+^. These patterns are quite different for glaucolide analogues where the base peak is the protonated molecule [M + H]^+^ whereas the in-source water loss peak is absent even when the compound bears free hydroxy groups. We applied the isolated compounds **1** and **7** to ESI-MS analysis with the aim to confirm these features (Figures S1–S2, supplementary data). Under the experimental conditions, the lactones **1** afforded a base peak at *m*/*z* 329.1 corresponding to the ion [M + H - H_2_O]^+^ together with the quasi-molecular ion [M+H]^+^ peak of low intensity at *m*/*z* 347.1 (6% RA) and the sodium adduct ion [M + Na]^+^ at *m*/*z* 369.1 (4% RA). 

**Figure 3:**
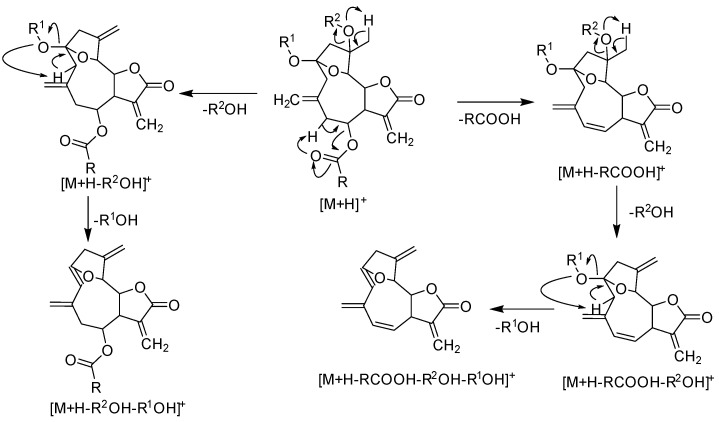
Proposed fragmentation patterns in hirsutinolide-like lactones under ESI conditions.

Compound **2**, also belonging to hirsutinolide-like lactones, showed an in-source loss of methanol [M + H - MeOH]^+^ as the most intense peak, while the proton and sodium adducts were less abundant. This variation in the fragmentation patterns of hirsutinolide-like lactones **1** and **2** could result from the methylation in **2** of the sole hydroxy group in **1** (Figure S3, supplementary data). On the other hand, the mass spectrum of compound **7** presented the quasi-molecular ion [M + H]^+^ at *m*/*z* 361.1 as the base peak, while the sodium adduct [M + Na]^+^ and the in-source water loss ion [M + H - H_2_O]^+^ were absent. The quasi-molecular ion peaks of the lactones **1** and **2** were accompanied by the fragmentation peak at *m*/*z* 243.1 also as the base peak. This ion might be generated from the loss of the methacrylic acid (MeacrOH, 86 Da) followed by elimination of H_2_O (18 Da) from **1** and MeOH from **2**. These patterns could help numbering hydroxy and methoxy groups in hirsutinolide-like lactones. Da Silva et al. (2020) also found similar fragmentation patterns when studying hirsutinolides under ESI conditions [9]. The fragmentation pattern is compiled in Figure 3.

The different main fractions from the crude extract were analyzed to detect potential non-isolated lactones of the extract. The LC-chromatogram of fraction B mainly showed four peaks (**I**–**IV**) (Figure S4, supplementary data). Peaks **I**–**II** and **III**–**IV** showed similar patterns to compound **1** for the former and to compound **2** for the latter meaning that **I**–**II** could be hirsutinolide-like lactones hydroxylated at C-2 while **III**-**IV** could correspond to methoxylated ones as previously described. The ESI spectra of compounds **1**–**3** were superimposable to that of peaks **I**, **III** and **II**, respectively. The unattributed peak I**V** (**1a**, Figure 2) has been tentatively considered as the methylated analogues of compound **2** at C-2, with the molecular formula C_21_H_26_O_6_ [13].

**Figure 4:**
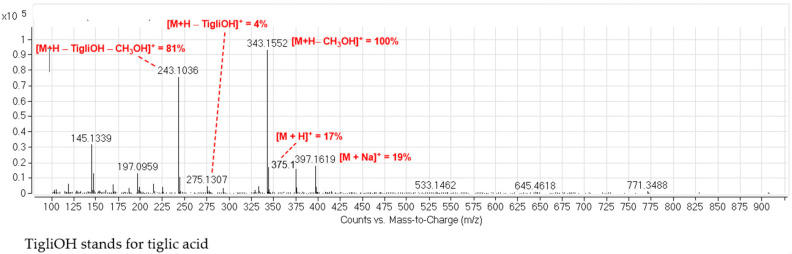
ESI-ToF-MS of peak **IV**.

In addition to the Na^+^ adduct at *m*/*z* 397.1 (19.1%), the LC-ESI-MS of peak **IV** showed the most stable peak at *m*/*z* 343.1 corresponding to an in-source loss of methanol [M + H - MeOH]^+^ and the quasi-molecular ion peak [M + H]^+^ with low intensity (17.0%) (Figure 4). Each of the peaks **I**–**IV** gave a fragment ion at *m*/*z* 243.1 characteristic for the above mentioned losses supporting the assignment of structures to peaks as proposed (Figure 3).

**Figure 5:**
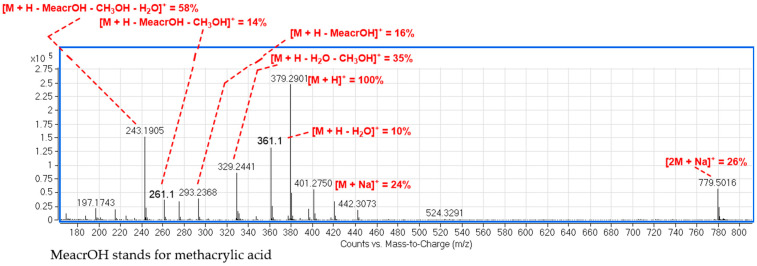
ESI-ToF-MS of peak **XIV**.

The sub-fraction P1C-3 from the main fraction P1C showed instead a panel of sesquiterpenoid lactones with similar patterns to compounds **1**–**2**. Compound **1** were present at *t*_R_ 8.317 min along with peaks **VI**–**XII** (Figure S5, supplementary data). Peak **IX** (compound **4**) generated a base peak at *m*/*z* 347.1 corresponding to the dehydrated quasi-molecular ion at *m*/*z* 365.1 (63% RA) together with the sodium adduct at *m*/*z* 387.1 (52% RA). The second in-source water loss [M + H − 2H_2_O]^+^ afforded a much less intense peak at *m*/*z* 329.1 (11% RA). The occurrence of two hydroxy groups in the structure of peak **IX** was confirmed by the fragment ions [M + H − MeacrOH]^+^ (*m*/*z* 279.1), [M + H − MeacrOH − H_2_O]^+^ (*m*/*z* 261.1) and [M + H−MeacrOH−2H_2_O]^+^ (*m*/*z* 243.1) resulting from successive losses of two water molecules from the parent ion generated by the loss of methacrylate group. Peak **XIV** in the LC chromatogram of sub-fraction P1C2-2P7 (Figure S6, supplementary data) showed instead successive losses of methanol and water from the demethacrylated ion [M + H − MeacrOH]^+^ (*m*/*z* 293.1, 16% RA) resulting in ions [M + H – MeacrOH − H_2_O]^+^ (*m*/*z* 275.1, 16.6% RA), [M + H − MeacrOH − CH_3_OH]^+^ (*m*/*z* 261.1, 14% RA) and [M + H − MeacrOH-H_2_O - MeOH]^+^ (*m*/*z* 243.1, 58% RA). Moreover, the quasi-molecular ion [M + H]^+^ also underwent similar fragmentations affording ions [M + H − H_2_O]^+^ (*m*/*z* 361.1, 10% RA) and [M + H − H_2_O - MeOH]^+^ (*m*/*z* 329.1, 35% RA) (Figure 5). Peak **XIV** could derive from a monomethylation of the analogous compound **4** since both ESI spectra were superimposable. Wu et al. (2017) reported the occurrence of this isomer in *E. mollis*, namely 8-*O*-methacryloylisoelephantane (**4a**), tentatively attributed to peak **XIV** [5]. Peak **VI** also showed as base peak the quasi-molecular ion [M + Na]^+^ at *m*/*z* 419.1 and with similar intensity the quasi-molecular ion [M + H]^+^ at *m*/*z* 397.1. Peak **VI** corresponds to the ion at *m*/*z* 379.1 [M + H − H_2_O]^+^ from the quasi-molecular ion [M + H]^+^ owing to the in-source water loss. However, the occurrence of the ion at *m*/*z* 293.1 from the protonated molecule suggests that the mass of the ester moiety should be 101 Da corresponding to hydroxymethacrylate, known to occur in most sesquiterpenoid lactones. We are not aware of any elephantopus-type lactones with this substituent so far. The molecule associated to peak **VI** could then be a new derivative. The decalin moiety of the molecule bears one hydroxy and one methoxy groups judged by fragment ions [M + H − MeacrOH − CH_3_OH]^+^ (*m*/*z* 261.1) and [M + H − MeacrOH − CH_3_OH − H_2_O]^+^ (*m*/*z* 243.1) (Figure S7, supplementary data). 

In contrast with ESI patterns of compounds **1**–**2**, peaks **VI**, **IX** and **XIV** showed the double molecule-Na^+^ complex ion [2M + Na]^+^ at *m*/*z* 815.2, 751.2 (32% RA) and 779.2 (26% RA), respectively. All these similarities, when combining ions occurring for hirsutinolides under ESI conditions and the double molecule sodium adduct ions, are indicative of similar core skeleton shared by peaks **VI**, **XIV** and compound **4** (peak **IX**). One could also notice a difference in relative abundances of protonated, sodium adduct and water loss ions when the hydroxy groups in elephantane-type skeleton, such as in **4** or **4a**, are free or substituted. This is the first report of elephantane-type lactone ESI-patterns that could serve as standards for the detection and characterization of hirsutinolide-like sesquiterpenoids in complex mixtures such as plant extracts using ESI-MS facilities. 

Nonetheless, peaks **VII** and **XII** both correspond to the quasi-molecular ion [M + Na]^+^ (*m*/*z* 415.1, **VII**) and *m*/*z* 383.1, **XII**) as the base peak along with the quasi-molecular ion [M+H]^+^, the ammonium adduct and the K^+^ adduct, respectively, at *m*/*z* 393.1 (25.8% RA), *m*/*z* 410.1 (23.6% RA) and *m*/*z* 431.1 (8.3% RA) for peak **VII** (Figure S8a, supplementary data) and *m*/*z* 361.1 (57.8% RA), *m*/*z* 378.1 (25.9% RA) and *m*/*z* 399.1 (4.4% RA) for peak **XII** (Figure S8b, supplementary data). Peak **VII** generated fragments at *m*/*z* 261.1 (78.3% RA) and (*m*/*z* 243.1, 80.4% RA) while peak **XII** fragmented into ions at *m*/*z* 275.1 (80.5% RA), 257.1 (15.1% RA) and 243.1 (12.4% RA). These fragmentation patterns were not evidenced in each of the isolated compounds used herein as standards and isolates within the frame of our study and could therefore represent new core skeletons of sesquiterpenoid lactones in *E. mollis*. Unfortunately, there is a lack of sesquiterpenoid ESI-patterns in the literature which could have help to complete the identification of these molecules.

Likewise, LC–MS data were exploited to detect and identify every caffeoyl derivatives of quinic acid occurring in *E. mollis*. The isolated analogues (**8**–**10**) were used as standards and their ESI–MS parameters were defined. Indeed, the DAD-chromatogram of P1E at 220–280 nm showed the occurrence of four UV-active peaks which could correspond to four caffeoyl derivatives (Figure S9, supplementary data). However, only three of them were isolated (**8**–**10**). The protonated monosubstituted quinic acid (**8**) [M + H]^+^ (*m*/*z* 355.1) was obtained as the base peak under the analysis conditions alongside the molecule-Na^+^ adduct at *m*/*z* 377.1 with relatively low intensity (10.8% RA) and the bimolecular-Na^+^ complex [2M + Na]^+^ at *m*/*z* 731.1 (11.0% RA). The in-source water loss molecule ion [M + H - H_2_O]^+^ at *m*/*z* 337.1 was almost unobserved (0.7% RA). For the disubstituted derivatives (**9**–**10**, **10a**), the base peaks change to the in-source dehydrated molecule [M + H - H_2_O]^+^ at *m*/*z* 499.1 accompanied by a relatively high (**10**, 84.3% RA and **10a**, 66% RA) or low (**9**, 20.0% RA) amount of the quasi-molecular ion at *m*/*z* 517.1. The sodium adduct ions were always present in very low abundance (1.5–2.2% RA) (Figure S9, supplementary data). However, considering the order of elution of dicaffeoyl isomers of quinic acid (diCA) on endcapped C18 columns available in the literature (3,5–diCA < 1,5−diCA < 4,5−diCA) the fourth peak was assigned to 4,5−diCA (**10a**) [25].

The main fraction P1E was constituted in large extent by carbohydrates as proven by the MS profile of peaks ranging from *t*_R_ 0.847–1.202 min (Figures S9–10, supplementary data) with quasi-molecular ions [M + Na]^+^ and [M + K]^+^ at *m*/*z* 203.1 and 219.1; at *m*/*z* 365.1 and 381.1, and at *m*/*z* 527.1 and 543.1, respectively. They correspond to a mono-, di- and trisaccharide tentatively attributed to glucose (**20a**) and sucrose (**21a**) for the first two, while the third one was not assigned. Compounds **20a** and **21a** were also identified by exploiting the NMR data of the mixture of these compounds. However, it was not possible to elucidate the structure of the trisaccharide (Figure 2).

### 2.2. Biological Endpoints of Compounds and Fractions

The crude extract, the flashed main fractions (P1A–P1E) and isolated compounds in high yields (**1**–**4**, **8**–**10**) were evaluated against the cervix carcinoma cell line KB-3-1. The crude extract showed low activity with IC_50_ > 100 mg/mL. The middle-polar fractions P1B and P1C were, however, more potent to inhibit the growth of the cell line with IC_50_ = 87 µM for P1B and IC_50_ = 21 µM for P1C compared to the reference drug griseofulvin (IC_50_ = 17–21 µM). Among the isolated compounds, only **2** and **3** were weakly active against KB-3–1 cells with IC_50_ = 22.6 µM for **2** and 45.2 µM for **3**.

Likewise, the samples were also screened against a panel of Gram-negative (*Escherichia coli* DSMZ 1058 and *Pseudomonas agarici* DSMZ 11810) and Gram-positive microorganisms (*Micrococcus luteus* DMSZ 1605, *Staphylococcus warneri* DSMZ 20036 and *Bacillus subtilis* DSMZ 704). The crude extract showed relatively low potential to inhibit the growth of the bacterial strains (DZI < 7 mm) at concentrations of 0.5 mg/mL. Nevertheless, and for the same concentrations, the middle-polar fractions P1B and P1C showed potent antibacterial properties against four of five strains tested including *P. agarici* (DZI = 15–16 mm), *M. luteus* (DZI = 13–15 mm), *S. warneri* (DZI = 10–11 mm) and *B. subtilis* (DZI = 14–15 mm). According to the MIC endpoints, P1B and P1C were moderately active against *P. agarici* and *B. subtilis* with MIC ranging from 25–54 µg/mL while the activity was low against *S. warneri* (MIC > 200 µg/mL) with 0.8–0.0015 mg/mL for each one. Conversely, fractions P1A and P1D showed moderate activity with DZI of 8 mm against *E. coli* and *B. subtilis* for P1A and DZI of 8–9 mm towards *B. subtilis* and *P. agarici* for P1D. The other strains were not sensitive to the tested samples including P1E. Our results were compared to those of gentamycin (reference drug), DZI = 17–21 mm at 0.5 mg/mL and MIC = 1.6–2.1 µg/mL. Likewise, only compound **2** showed a low activity against *P. agarici* DSMZ 11810 (DZI = 9 mm), *M. luteus* DMSZ 1605 (DZI = 7 mm) and *B. subtilis* DSMZ 704 (DZI = 8 mm). The isolated compounds were also screened for their scavenging effects against the radical DPPH. Amongst the tested samples, only compounds **8** and **10** displayed moderate activity with IC_50_ of 118 and 34 µg/mL, respectively, compared to the reference drug trolox (IC_50_ = 20 µg/mL). The absence of activity of compound **9** could be related to its insolubility in DMSO used for these assays as solvent. 

## 3. Discussion

To date, more than 20 sesquiterpenoid lactones have been reported from *Elephantopus mollis* worldwide [1,4,5,13,15,26]. Chemophenetic uses chemical ingredients to define new clades that were previously not recognized or cannot be defined based on morphology [27]. In general, germacranolides and guaianolides are the chemophenetic markers of the studied plant genus [1,4,5,13,15,26,28]. Nine of them were identified or detected in the frame of our study, while compound **3** is being reported for the first time in the species and even in the genus *Elephantopus*. When comparing Cameroonian and Chinese species, one could highlight that seven of the lactones encountered so far were unique in the Chinese species while three were present only in the Cameroonian variant [5,13,15]. The Chinese species highlighted derivatives where substituents such as ethoxy at C-4 in compound **4** or angelate or tiglate at C-8 in compound **6** instead of hydroxy and acrylate, respectively, and a second lactone moiety due to the oxidized C-15/C-2 positions or the methoxylation of the *α*-methylene butyrolactone in **1** and **2** [5]. Some sesquiterpenoid skeletons already relayed in the literature such as guaianolide-type sesquiterpene lactones were not encountered in the present study. Our study is also highlighting the occurrence of an elanane-type sesquiterpene, compound **16**, in the genus *Elephantopus*. This class of sesquiterpenes has been reported in some species of the Asteraceae plant family including *Vernonia*. 

Our study also points out the occurrences of phenolic acids (**14**–**15**), flavonoids (**11**–**13**) and quinic acid derivatives (**8**–**10**) in *Elephantopus* species. Apart from compound **10** reported in *E. mollis* and compounds **8**, **11** and **12** reported in *E. scaber*, each of these phytoconstituents are herein reported for the first time in the genus *Elephantopus* [28,29]. Methoxylated flavonoids are abundant in Asteraceae species while dicaffeoylquinic acids are scarce [24]. The occurrences of phenolic acids in *E. mollis* could be related to the presence of quinic acid since the latter is an intermediate in the biosynthesis of the former. Some chemophenetic markers of Asteraceae plant species namely the steroids **17**, the carbohydrates **21a** and the monoglyceride **19**, were also elucidated [24]. The monoglyceride was isolated for the first time in the studied genus. 

Overall, the samples were moderately active against both bacterial strains and cancer cell line used. The studied plant and sesquiterpene lactones are reputed for their anticancer and anti-inflammatory activities because of the *α*-methylene-*γ*-butyrolactone moiety in their structures [5,6,8,13,15]. The MeOH extract of *E. mollis* has been reported to exhibit significant activity against a panel of colorectal, liver and breast carcinoma [30]. We report herein for the first time the cytotoxicity of the plant towards the cervix carcinoma cell line KB-3-1. The results obtained from the middle-polar main fractions P1B and P1C were similar to those reported for the EtOAc extract of *E. mollis* against the liver carcinoma HepG2 [3]. Compounds **2**, **4a**, **3** and **7** have been reported to be active against leukemia K562 and HL−60 and neuroblastoma B104 cell lines with IC_50_ less than 5 µM [13,15,31]. As for the extract, this is the first report on the cytotoxicity of both main fractions and sesquiterpenoid lactones against the cancer cell lines KB-3-1. Likewise, we highlight here for the first time the antibacterial activity of the samples against the tested strains.

Nonetheless, only disubstituted quinic acid shows antiradical scavenging effect toward DPPH probably due to caffeoyl moieties as indicated in the literature. The radical scavenging activity in the chlorogenic series increased with the number of caffeoyl substituents. Compound **10** was the most active antioxidant constituent as reported in the literature [28]. The other sesquiterpenes and compounds were not active. Our results confirm readily data available in the literature on the study plant *E. mollis* [13,15,26,32]. The active constituents of this plant have been identified to sesquiterpenoid lactone which is known to bind sulfhydryl-containing enzymes and proteins in pathogens resulting in a programmed death of organisms [13,15,26,32]. Apart from their antioxidant capacities, caffeoyl derivatives of quinic acids are well-known for their antiviral potency and extracts of *E. scaber* have been already patented to alleviate virus form of symptoms of diseases [33].

## 4. Materials and Methods

### 4.1. General Experimental Procedures

Electrospray ionization (ESI) mass spectra were recorded on a 1200-series HPLC-system or a 1260-series Infinity II HPLC-system (Agilent-Technologies) with binary pump and integrated diode array detector coupled to an LC/MSD-Trap-XTC-mass spectrometer (Agilent-Technologies) or an LC/MSD Infinity lab LC/MSD (G6125B LC/MSD). High resolution mass spectra were recorded on a Micromass-Q-ToF-Ultima-3-mass spectrometer (Waters) with LockSpray-interface and a suitable external calibrant. The mobile phase used for the LC–MS comprised gradients of acetonitrile-water (containing 0.1% formic acid). UV–Vis spectra were recorded on an Evolution 201 UV–Visible Spectrophotometer (Thermo Scientific) and infrared (IR) spectra on a FTIR-spectrometer (Bruker Tensor 27) equipped with a diamond ATR unit and are reported in terms of frequency of absorption in cm^–1^. NMR spectra were recorded on a Bruker Avance III 500 HD (^1^H: 500 MHz, ^13^C: 126 MHz) or Avance 600 (^1^H: 600 MHz, ^13^C: 151 MHz). Chemical shifts *δ* (ppm) are reported relative to residual solvent signal and/or tetramethylsilane (TMS). 2D spectra (COSY, HMQC, HMBC) and DEPT-135 spectra were used for signal assignment.

Chromatographic purification of compounds was performed on silica gel (35–70 μm, Acros Organics) and Sephadex LH-20. Automated column chromatography was also performed on a Büchi Reveleris^®^ X2 with a binary pump and ELSD Detector using flash up column of 4 g, 12 g and 24 g, depending on the sample mass, or a Biotage Snap Ultra C18 column with a gradient at various flow rate. Thin-layer chromatography (TLC) was carried out on silica plates (TLC Silica 60 F_254_ by Merck) and spots were detected by spraying with 20% H_2_SO_4_ followed by charring at 100 °C. 

Analytical LC-MS was performed on an Agilent 6220 ToF-MS with a Dual ESI-source, 1200 HPLC system with autosampler, degasser, binary pump, column oven, diode array detector and a Hypersil Gold C18 column (1.9 µm, 50 x 2.1 mm) with a gradient (in 11 min from 0%B to 98%B, back to 0%B in 0.5 min, total run time 15 min) at a flow rate of 300 µL/min and column oven temperature of 40 °C. HPLC solvent A consists of 94.9% water, 5% acetonitrile and 0.1% formic acid, solvent B of 5% water, 94.9% acetonitrile and 0.1% formic acid. The mass spectra are recorded in both profile and centroid mode with the MassHunter Workstation Acquisition B.04.00 software (Agilent Technologies, Santa Clara, CA, USA). MassHunter Qualitative Analysis B.07.00 software (Agilent Technologies, Santa Clara, CA, USA) was used for processing and averaging of several single spectra.

### 4.2. Plant Material

The whole plant of *Elephantopus mollis* was collected on the savanna hills of Bamendjing (Mbouda) in the western Region of Cameroon in December 2019. The identification was performed by Mr. Victor Nana, an experienced botanist, at the Cameroon National Herbarium (Yaoundé) by comparison with the voucher specimen kept under the voucher number 35121/HCN. 

### 4.3. Extraction and Purification

Dried materials of *Elephantopus mollis* (1.0 kg) were ground and macerated with methanol (3 x 5 L, 72 h each) at room temperature to yield a semi-solid crude extract (110.0 g) after removal of the solvent under reduced pressure on a rotary evaporator. A portion (100.0 g) of this extract was flashed over an open silica gel column chromatography with gradients of Et_2_O-acetone then acetone-MeOH. In this case, 70 fractions of 200 mL each were collected and combined based on their TLC profiles (using mixtures of Et_2_O-acetone 85:15, 70:30, 30:70) into five main fractions coded P1A-P1E (P1A: 1–8; P1B: 9–18; P1C: 19–40; P1D: 41–53; P1E: 54–70). Fraction P1A (5.1 g) contained mostly lipids and was not further investigated. Fraction P1B (4.0 g) was flashed on a normal phase middle pressure liquid chromatography (NP-MPLC) with gradients of Et_2_O-EtOAc. Compound **3** was eluted at 77–80 min. Fraction P1C (1.5 g) was flashed over an open silica gel column chromatography with gradients of Et_2_O-acetone and yield 100 fractions of 100 mL each, collected and combined base on their TLC profiles (using mixtures of Et_2_O-acetone 85:15, 70:30, 30:70) into four main sub-fractions coded P1C1–P1C5. Each of these fractions was flashed on a NP-MPLC with gradients of Et_2_O-EtOAc. P1C1 led to the isolation of compound **19**. P1C2 gave rise to compounds **1** at *t*_R_ 7–8 min, **15** at *t*_R_ 10 min and **16** at *t*_R_ 11-15 min while the remaining mixtures were each also flashed under the same conditions with NP-MPLC. Compound **14** was eluted at *t*_R_ 12 min; the mixture of **6** and **7** at *t*_R_ 40–43 min and compound **4** at *t*_R_ 53–55 min. The same method applied to the remaining sub-fractions was not conclusive. Fraction P1D was treated as was P1C through an open column chromatography with gradients of Et_2_O-acetone prior to NP-MPLC purification with a gradient of DCM-EtOAc. Amongst the main sub-fractions collected, only P1D-3 were conclusive affording compounds **2** and **11**–**13**. The steroids **17** and **18** were eluted from the open-column chromatography of P1D. Fractions P1E separated on an open silica gel column chromatography with gradients of DCM-MeOH yielded four main sub-fractions P1E1-P1E4. P1E1 and P1E2 gave relatively same constituents as P1D. P1E3 was further chromatographed on a reversed-phase MPLC with gradients of acidified ACN-H_2_O. Compounds **8**–**10** were eluted at *t*_R_ 47, 49 and 57 min, respectively. The remaining compounds (**1a**, **4a**, **10a**, **20a**, **21a**) were detected and analyzed on LC–MS profiles.

### 4.4. Bioactivity

The antibacterial activity of some isolated compounds was carried out by using the agar disk diffusion method to determine the diameter zone of inhibition (DZI) against five bacteria including two Gram-negative (*Escherichia coli* DSMZ 1058 and *Pseudomonas agarici* DSMZ 11810) and three Gram-positive (*Micrococcus luteus* DMSZ 1605, *Staphylococcus warneri* DSMZ 20036 and *Bacillus subtilis* DSMZ 704) strains [10,11]. The microorganisms were provided by DSMZ (German Collection of Microorganisms and Cell Cultures).

The cytotoxicity assay was performed with the human cervix carcinoma cell line KB-3-1 as previously reported [10,11]. The KB-3-1 cells were provided by DSMZ (number ACC 158) and cultivated as a monolayer in DMEM (Dulbecco’s modified Eagle medium) with glucose (4.5 g/L), L-glutamine, sodium pyruvate and phenol red, supplemented with 10% (KB-3-1) foetal bovine serum (FBS). On the day before the test, the cells (70% confluence) were detached with trypsin-ethylenediamine tetraacetic acid (EDTA) solution (0.05%; 0.02% in DPBS) and placed in sterile 96-well plates in a density of 10,000 cells in 100 μL medium per well. The dilution series of the compounds were prepared from stock solutions in DMSO of concentrations of 1 mM or 10 mM. The stock solutions were diluted with culture medium (10% FBS) at least 50 times. Some culture medium was added to the wells to adjust the volume of the wells to the wanted dilution factor. The dilution prepared from stock solution was added to the wells and each concentration was tested in six replicates. The control contained the same concentration of DMSO as the first dilution. After incubation at 37 ºC and 5.3% CO_2_-humidified air for 72 h, 30 μL of an aqueous resazurin solution (175 μM) was added to each well. The cells were incubated for 6 h at the same conditions. Thereafter, the fluorescence was measured. The excitation was affected at a wavelength of 530 nm, whereas the emission was recorded at 588 nm. The IC_50_ that is the values equal the drug concentrations, at which vitality is 50%, were calculated as a sigmoidal dose response curve using GRAPHPAD PRISM 4.03. The antioxidant activity of compounds using DDPH was determined according to the methods described by Nguemo et al. 2020 [34].

## Data Availability

The raw data supporting the conclusions of this article will be made available by the authors, without undue reservation, to any qualified researcher

## Supplementary Materials

LC chromatograms and MS spectra (Figures S1–S10) as well as NMR spectra of standards listed in the text (Figures S11–S36) are available as supporting information. Figure S1: ESI-TOF-MS of compound 1., Figure S2: ESI-TOF-MS of compound 7., Figure S3: ESI-TOF-MS of compound 2., Figure S4: LC chromatogram of main fraction B., Figure S5: LC profile of subfraction C3 and ESI-TOF-MS of compound 4., Figure S6. LC chromatogram of sub-fraction P1C-C2-2P7., Figure S7. ESI-TOF-MS of peak VI., Figure S8. LC chromatogram of (a) peak VII and (b) peak XII., Figure S9. LC chromatogram of quinic acid derivations 8–10 and 22., Figure S10. LC chromatogram of crude extract P1E and MS profiles of sugars, Figure S11. MPLC chromatogram of fraction P1B., Figure S12. MPLC chromatogram of fraction P1C2., Figure S13. MPLC chromatogram of sub-fraction P1C2-2., Figure S14. MPLC chromatogram of sub-fraction P1E3.

## Sample Availability

Samples of the compounds are not available from the authors.
