# Supplementary material for: LC-ToF-ESI-MS Patterns of Hirsutinolide-like Sesquiterpenoids Present in the Elephantopus mollis Kunth Extract and Chemophenetic Significance of Its Chemical Constituents
Source: Molecules. 2021 Aug 9;26(16):4810. doi: 10.3390/molecules26164810 (PMC8399899; doi:10.3390/molecules26164810)
Supplement: Supplementary file 1 [file molecules-26-04810-s001.zip › molecules-1296789-supplementary.pdf]

molecules

MDPI

Supplementary materials

LC-TOF-ESI-MS Patterns of Hirsutinolide-like Sesquiterpenoids from *Elephantopus mollis* Kunth Alongside Chemophenetic significance of its chemical constituents

Gabin Thierry M. Bitchagno[1,2,*], Jean Garba Koffi[3], Ingrid Konga Simo[1], Donald Ulrich K. Kagho[4], Augustin Silvere Ngouela[1], Bruno Ndjakou Lenta[3,*] and Norbert Sewald[2]

[1] Faculty of Sciences, Department of Chemistry, University of Dschang, Cameroon
[2] Organic and Bioorganic Chemistry, Faculty of Chemistry, Bielefeld University, D-33501, Bielefeld, Germany
[3] Department of Chemistry, Higher Teacher Training College, University of Yaounde I, P. O. Box 47, Yaounde, Cameroon
[4] Department of Organic Chemistry, Faculty of Science, University of Yaoundé 1, P.O. Box 812, Yaounde, Cameroon
* Correspondence: **authors:**Prof. Dr. Bruno Ndjakou Lenta, Department of Chemistry, Higher Teacher Training College, University of Yaounde I, P. O. Box 47, Yaounde, Cameroon. Tel. 675097561; E-mail: lentabruno@yahoo.fr;Dr Gabin Thierry M. Bitchagno, Department of Chemistry, University of Dschang, P.O. Box 67, Dschang, Cameroon. Tel: +237 674098757; E-Mail: gabin1256@gmail.com

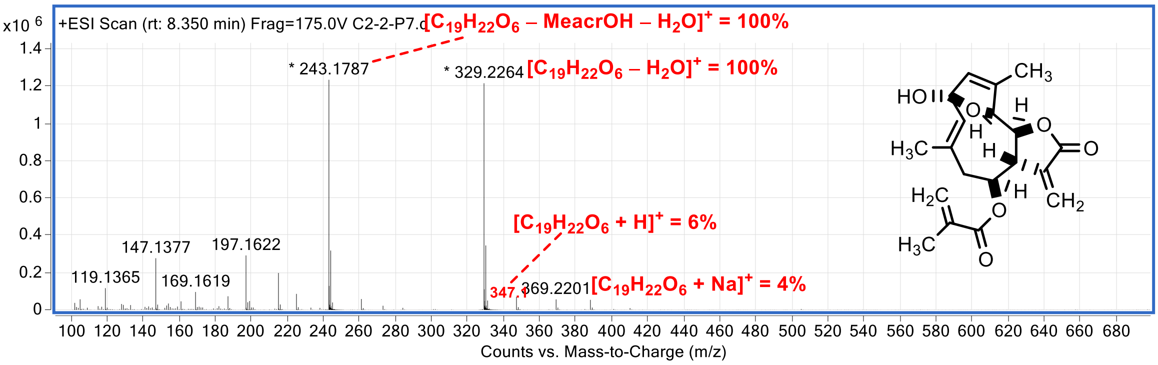

Figure S1. ESI-TOF-MS of compound **1.**

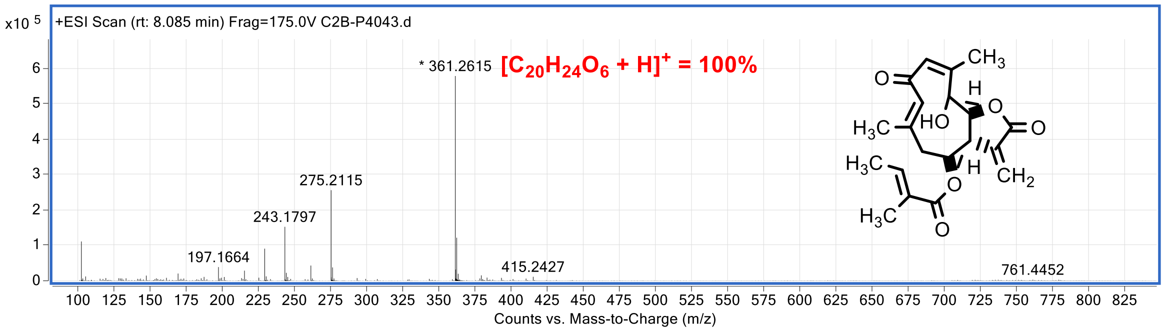

Figure S2. ESI-TOF-MS of compound **7.**

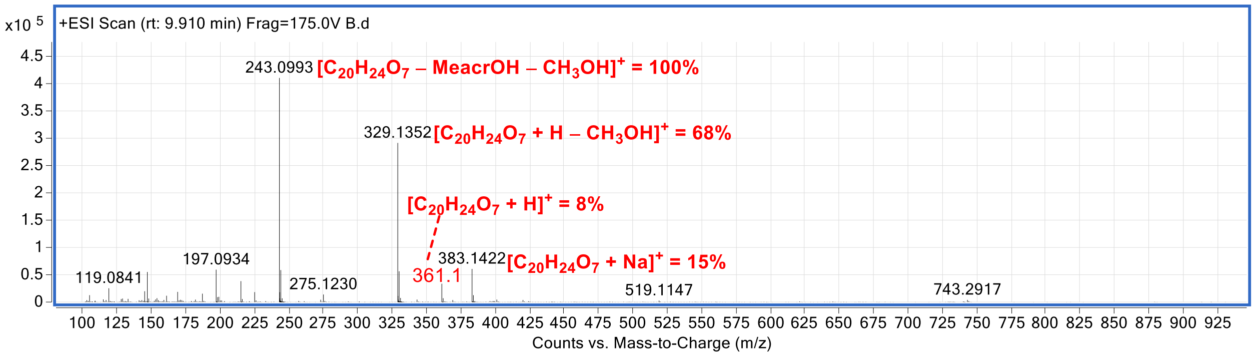

Figure S3. ESI-TOF-MS of compound **2.**

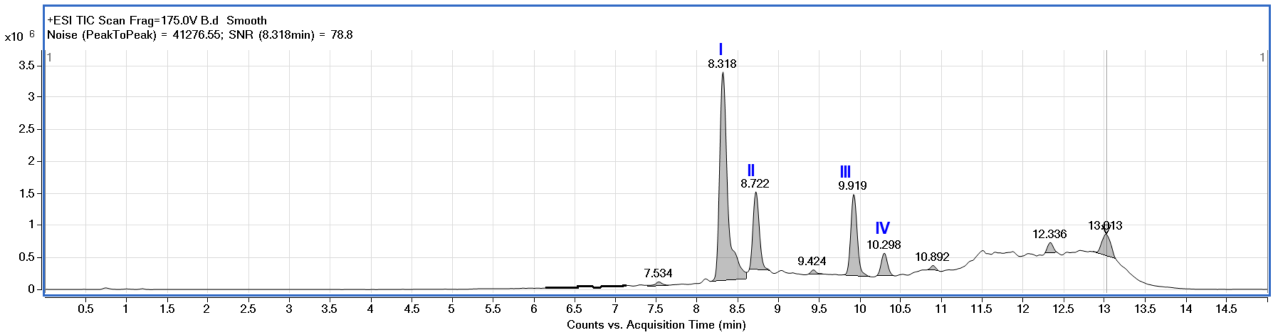

Figure S4. LC chromatogram of main fraction B.

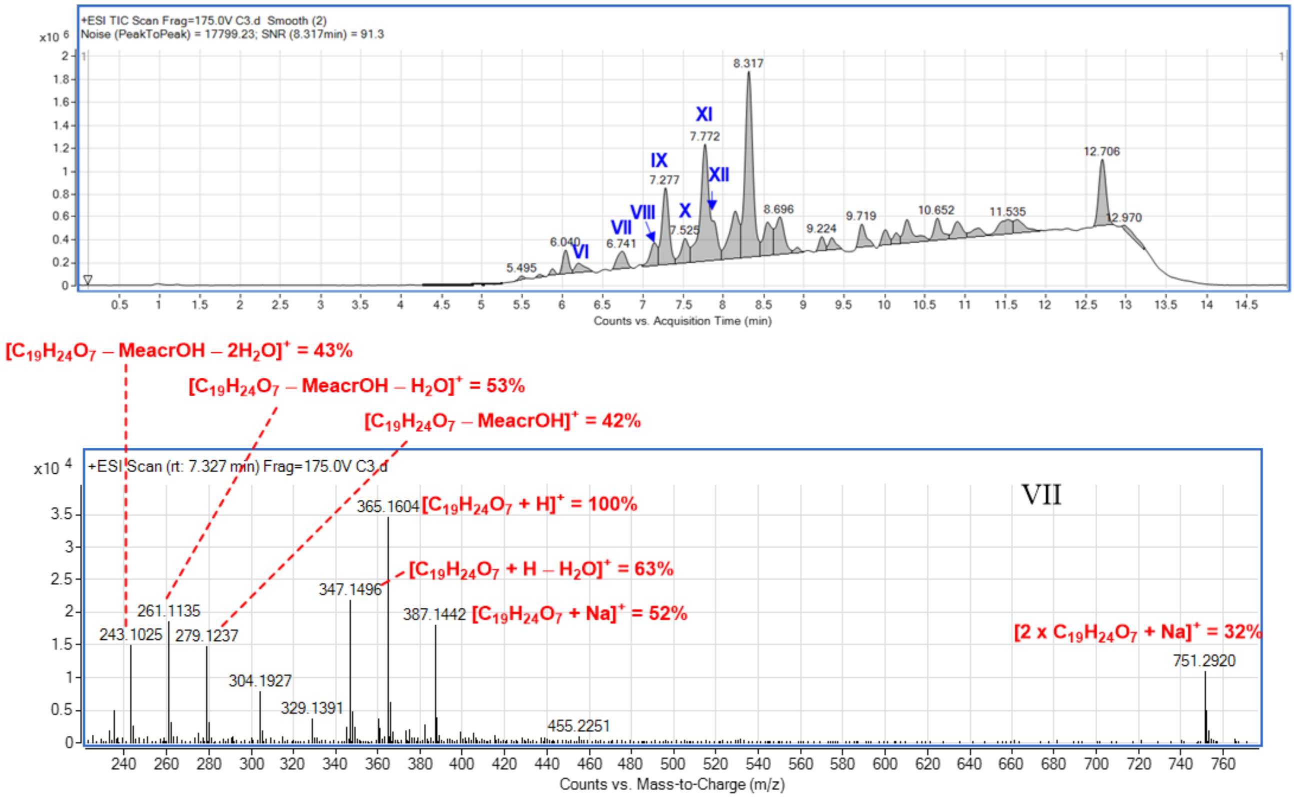

Figure S5. LC profile of subfraction C3 and ESI-TOF-MS of compound **4.**

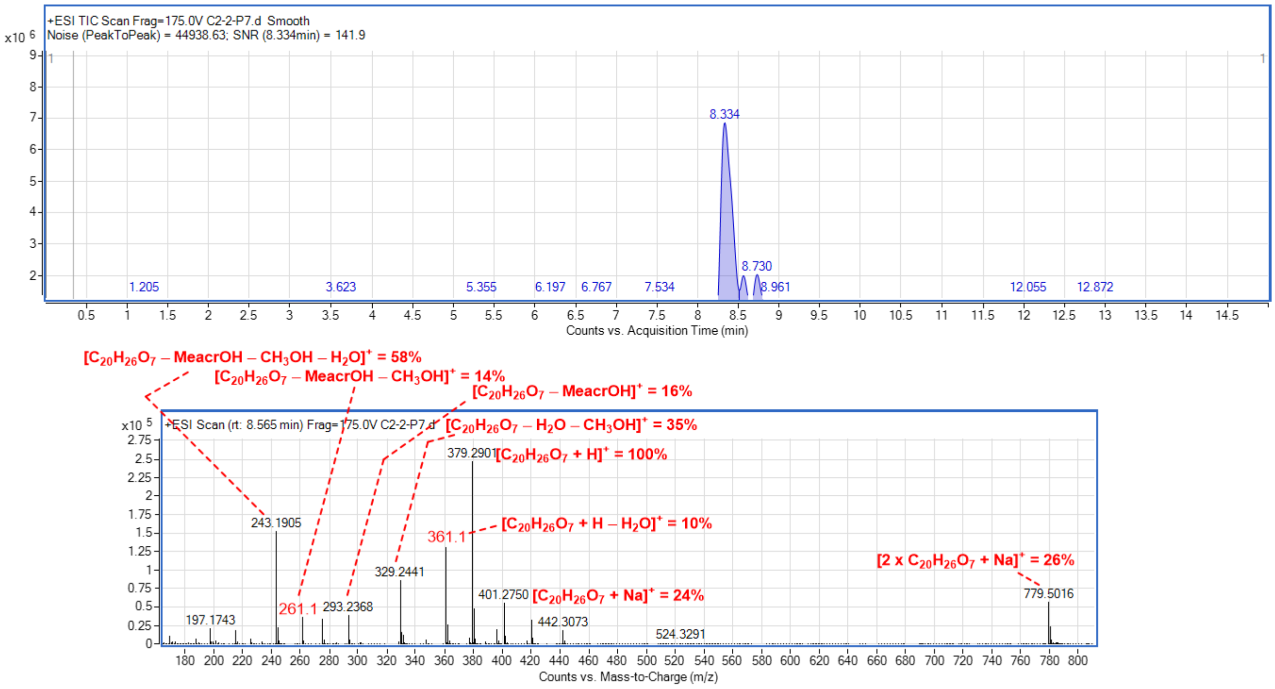

Figure S6. LC chromatogram of sub-fraction P1C-C2-2P7.

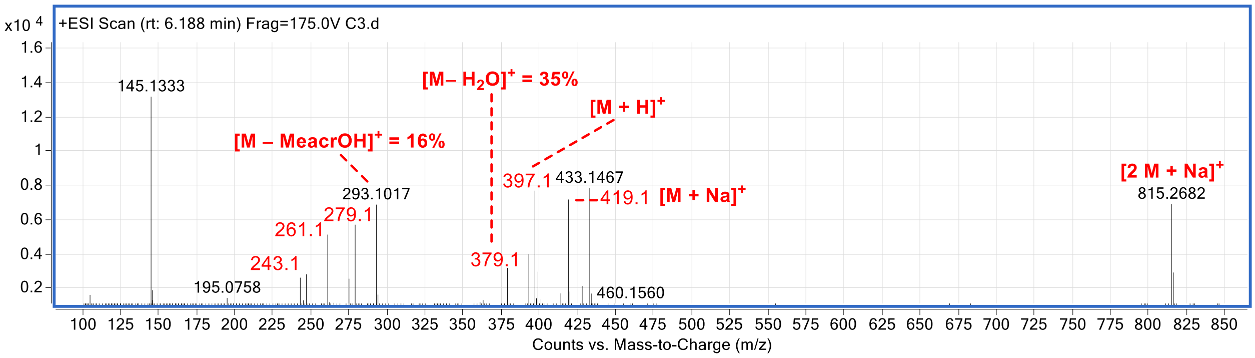

Figure S7. ESI-TOF-MS of peak **VI.**

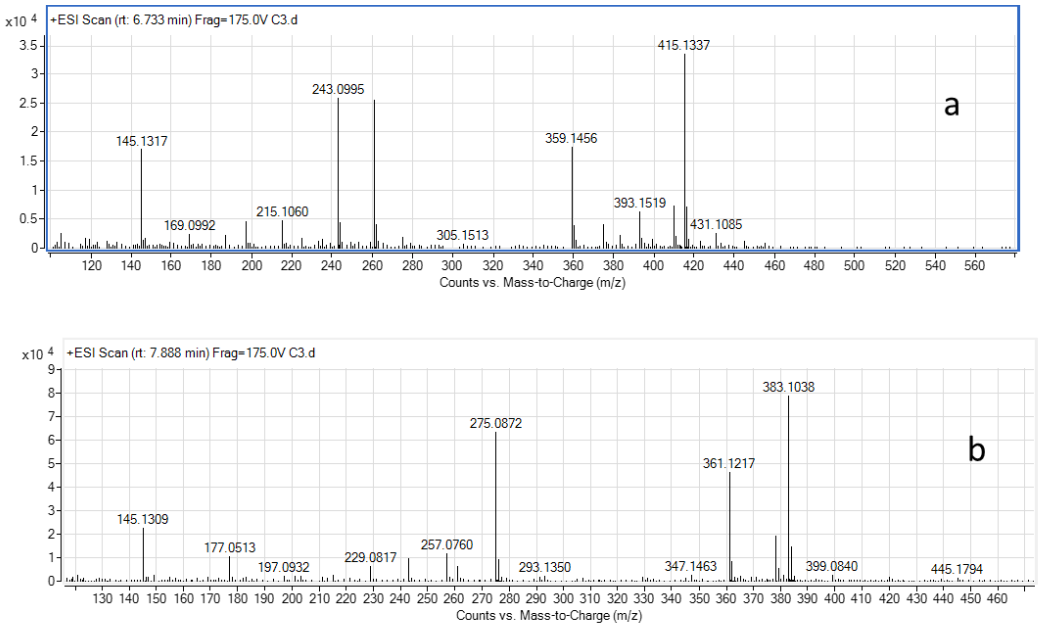

Figure S8. LC chromatogram of (a) peak VII and (b) peak XII.

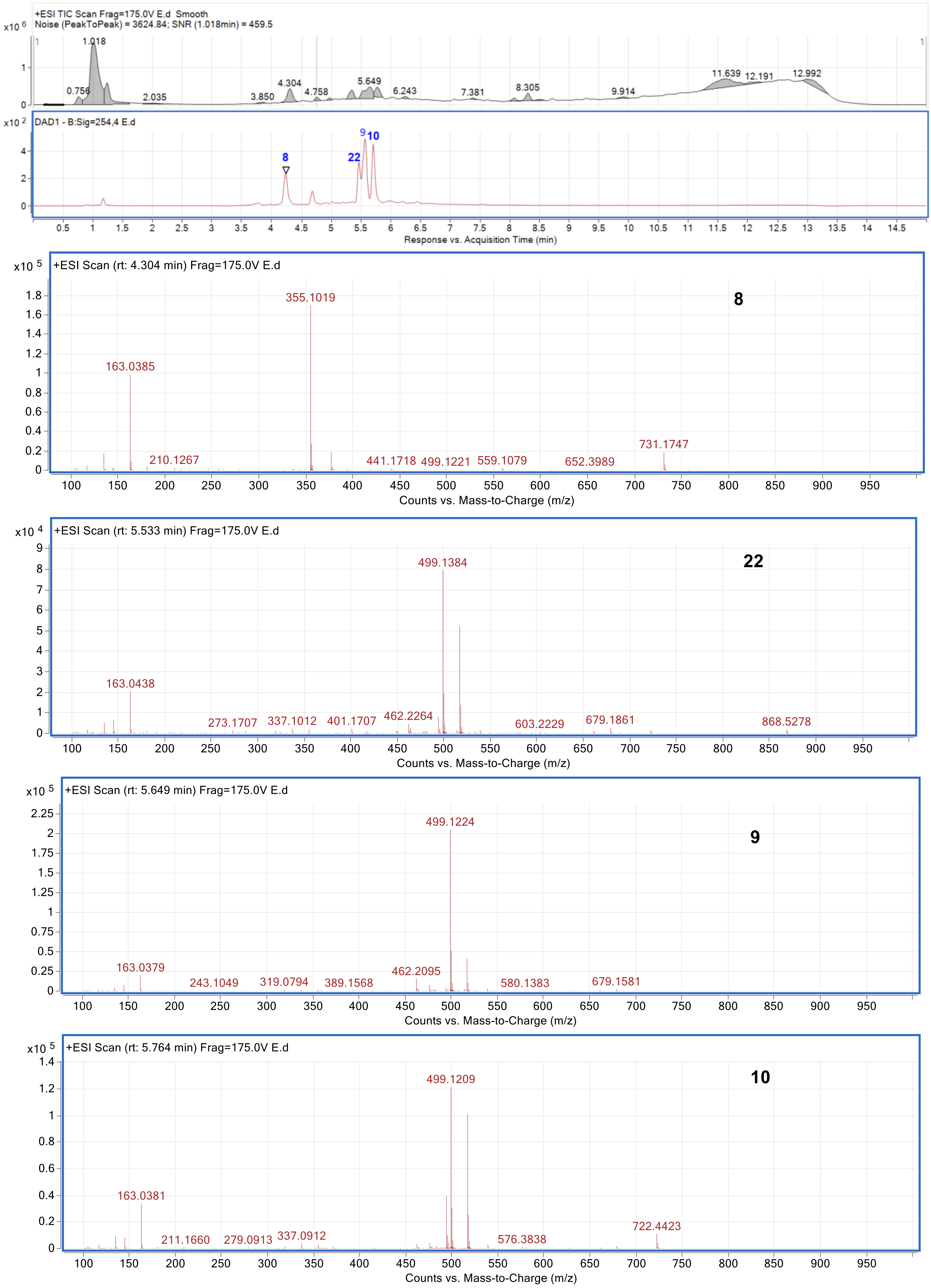

Figure S9. LC chromatogram of quinic acid derivations **8-10** and **22.**

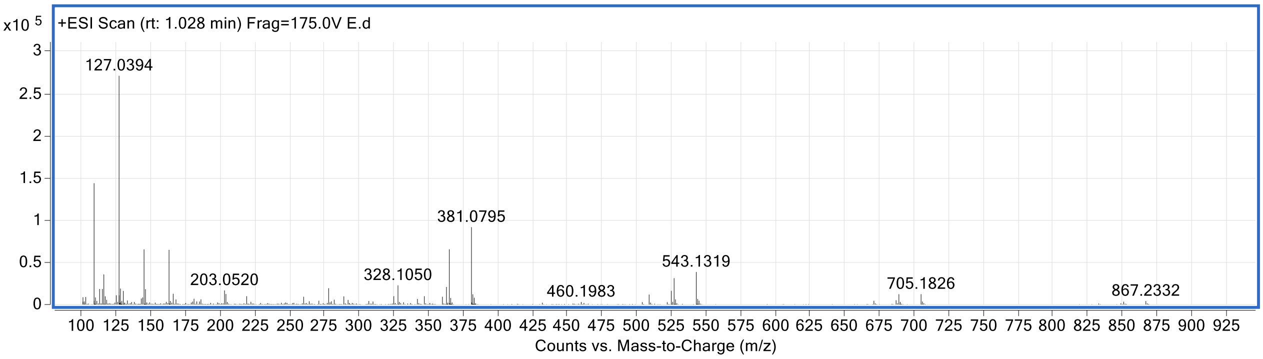

Figure S10. LC chromatogram of crude extract P1E and MS profiles of sugars.

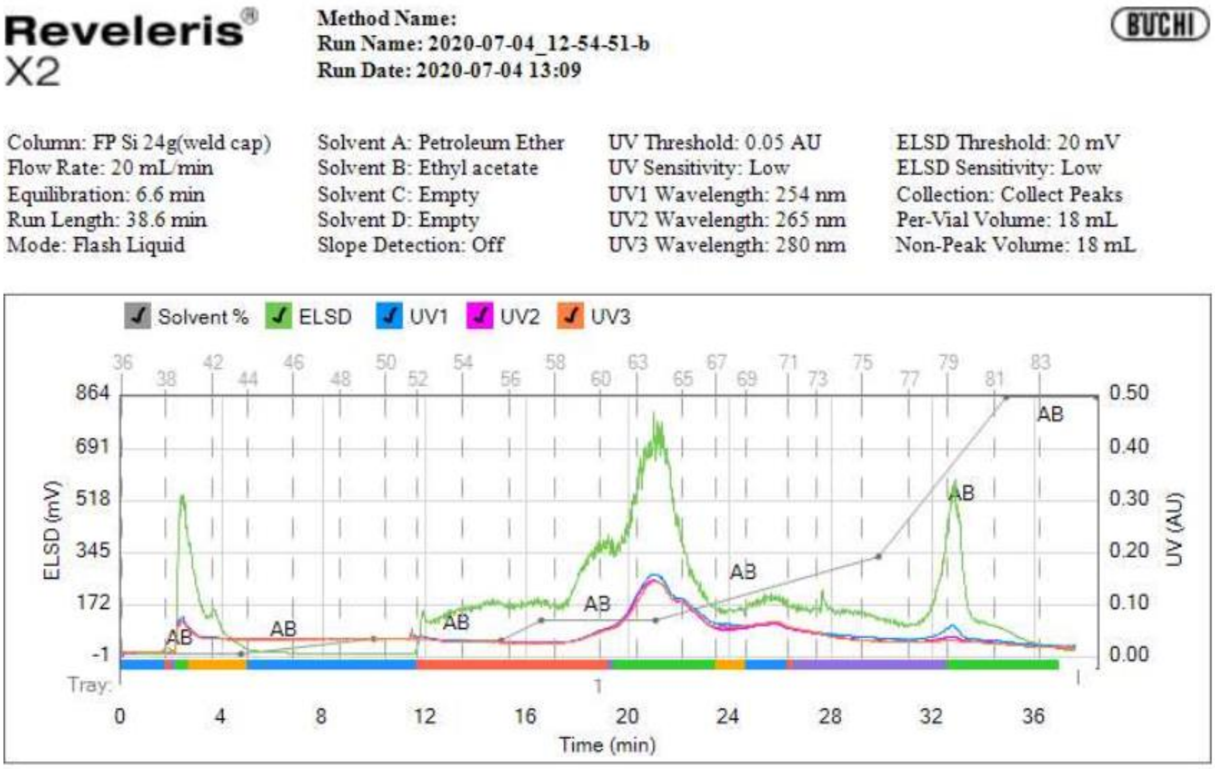

Figure S11. MPLC chromatogram of fraction P1B.

Reveleris® X2

Method Name:
Run Name: 2020-05-23_14-43-26-c2-bleue
Run Date: 2020-05-23 14:57

BÜCHI

Column: FP Si 24g(weld cap)	Solvent A: Dichloromethane	UV Threshold: 0.05 AU	ELSD Threshold: 20 mV
Flow Rate: 32 mL/min	Solvent B: Ethyl acetate	UV Sensitivity: Low	ELSD Sensitivity: Low
Equilibration: 6.6 min	Solvent C: Empty	UV1 Wavelength: 254 nm	Collection: Collect All
Run Length: 45.0 min	Solvent D: Empty	UV2 Wavelength: 265 nm	Per-Vial Volume: 18 mL
Mode: Flash Liquid	Slope Detection: Off	UV3 Wavelength: 280 nm	Non-Peak Volume: 18 mL

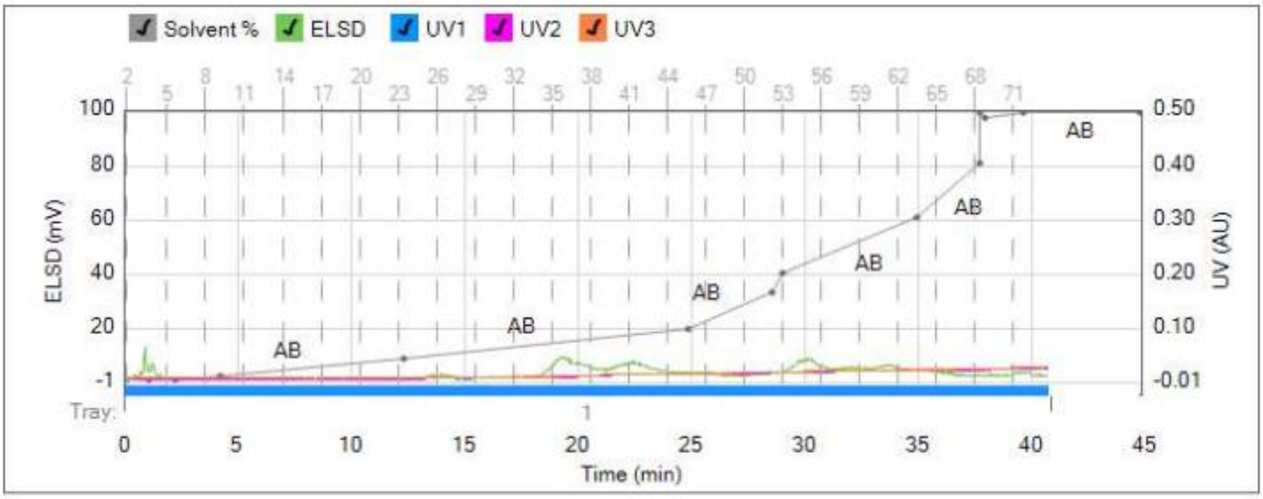

Figure S12. MPLC chromatogram of fraction P1C2.

Reveleris® X2

Method Name:
Run Name: 2020-03-13_08-03-10-p1-c2-2
Run Date: 2020-03-13 08:14

BÜCHI

Column: FP Si 24g(weld cap)	Solvent A: Petroleum Ether	UV Threshold: 0.05 AU	ELSD Threshold: 20 mV
Flow Rate: 32 mL/min	Solvent B: Ethyl acetate	UV Sensitivity: Low	ELSD Sensitivity: Low
Equilibration: 4.0 min	Solvent C: Empty	UV1 Wavelength: 254 nm	Collection: Collect Peaks
Run Length: 22.0 min	Solvent D: Empty	UV2 Wavelength: 265 nm	Per-Vial Volume: 18 mL
Mode: Flash Liquid	Slope Detection: Off	UV3 Wavelength: 280 nm	Non-Peak Volume: 18 mL

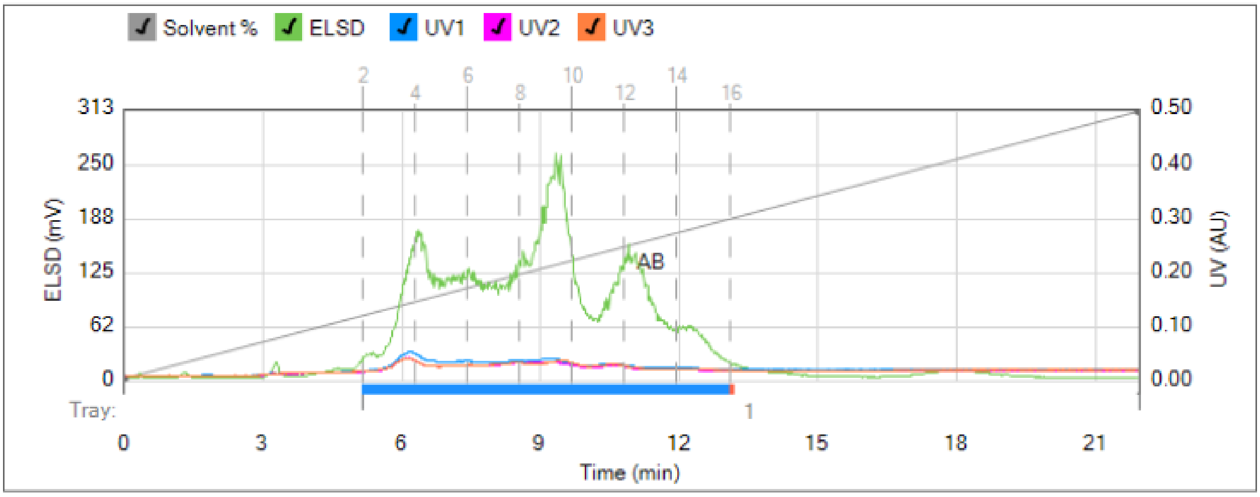

Figure S13. MPLC chromatogram of sub-fraction P1C2-2.

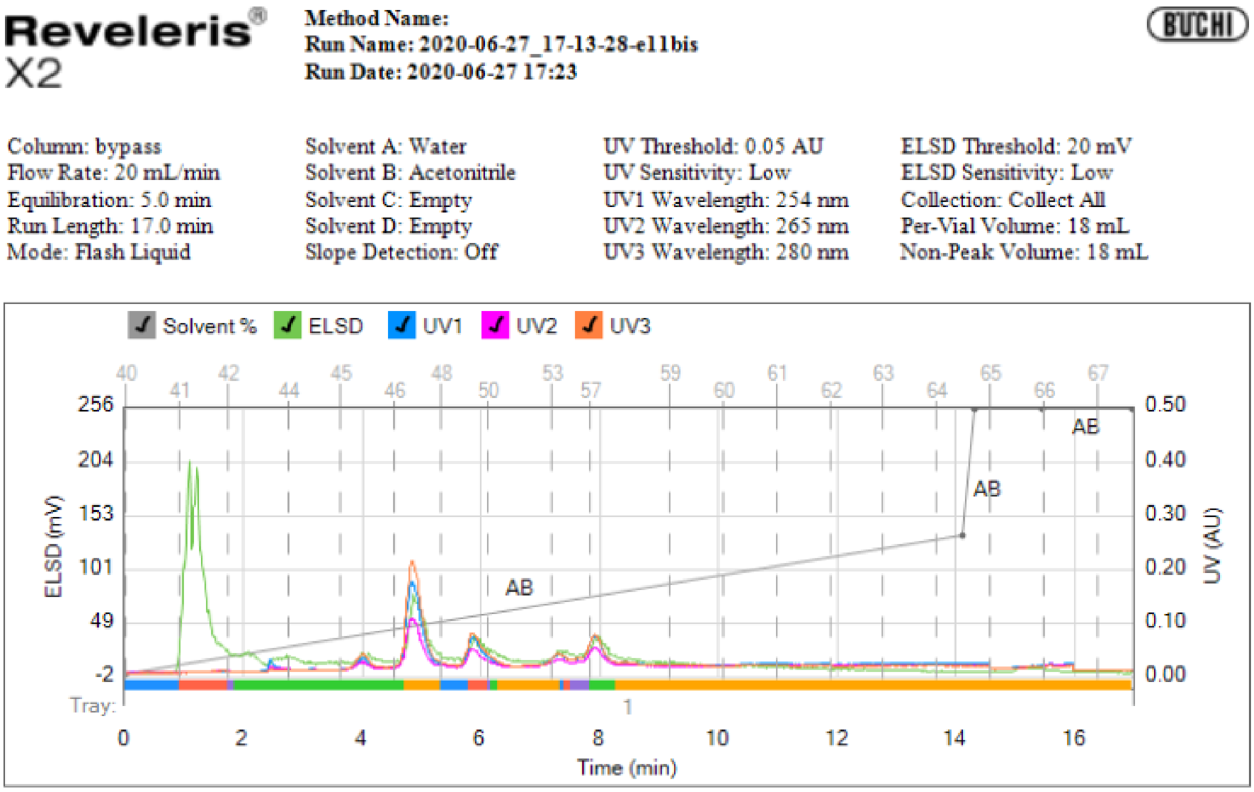

Figure 14. MPLC chromatogram of sub-fraction P1E3.
